# The Temporal and Spatial Distribution Patterns of Necrotic and Apoptotic Cells in and Around the Spinal Cord Injury Site †

**DOI:** 10.3390/diagnostics15162067

**Published:** 2025-08-18

**Authors:** Selim Ayhan, Gokhan Bozkurt, Atilla Akbay, Mutlu Hayran, Hiroshi Ogawa, Wataru Yasui, Masato Tanaka, Ayse Ayhan

**Affiliations:** 1 Department of Neurological Surgery, Baskent University School of Medicine, Ankara 06490, Turkey; 2 Department of Neurological Surgery, Hacettepe University School of Medicine, Ankara 06100, Turkey; atik@hacettepe.edu.tr; 3 Department of Neurological Surgery, Acibadem Maslak Hospital, Istanbul 34457, Turkey; 4 Department of Neurological Surgery, Bayindir Sogutozu Hospital, Ankara 06530, Turkey; 5 Department of Preventive Oncology, Hacettepe University Institute of Oncology, Ankara 06100, Turkey; mhayran@hacettepe.edu.tr; 6 Department of Pathology, Seirei Mikatahara General Hospital, Hamamatsu 433-8558, Japan; hirogawa@gmail.com; 7 Department of Molecular Pathology, Institute and Graduate School of Biomedical & Health Sciences, Hiroshima University, Hiroshima 734-8553, Japan; wyasui@hiroshima-u.ac.jp; 8 Department of Orthopedic Surgery, Okayama Rosai Hospital, Okayama 702-8055, Japan; tanaka0896@gmail.com; 9 Department of Tumor Pathology, Hamamatsu University School of Medicine, Hamamatsu 431-3192, Japan; 10 Molecular Genetics Laboratory of Female Reproductive Cancer, Johns Hopkins University School of Medicine, Baltimore, MD 21231, USA

**Keywords:** spinal cord injury, apoptosis, necrosis, mapping, ssDNA, rat

## Abstract

**Background**: Acute spinal cord trauma management necessitates understanding the primary and secondary injury mechanisms at different timepoints. **Objectives**: To characterize the cell death process by examining the temporal and spatial distributions of necrosis and apoptosis in an experimental spinal cord injury model. **Methods**: Wistar male rats were divided into trauma (n = 30) and sham (n = 6) groups, and a 50 g/cm weight drop contusion design was used. The rats were sacrificed 1, 6, 24, 48, 72, and 168 h after the injury. Every 0.5 cm spinal cord segment was examined cranially and caudally up to a total of 2.5 cm for neuronal and glial damage via the apoptotic count and DNA damage index via morphology and immunohistochemistry using an anti-ssDNA antibody. The results were mapped to visualize the damage extent, intensity, and distribution. **Results**: The central zone underwent hemorrhage and necrosis one hour after the injury. The apoptotic cells and DNA damage index increased with time (*p* < 0.001), and specific spatial alterations were observed among the segments (*p* < 0.001). Mapping the apoptotic cells and DNA damage clearly reflected the injury’s severity and extent. **Conclusion**: The DNA damage and the apoptotic cell count increase over time were well correlated with the morphology and could easily be elucidated using ssDNA immunostaining.

## 1. Introduction

Acute traumatic spinal cord injury (SCI) remains a major medical and socioeconomic challenge, with an incidence of 15–40 per million annually [1,2,3]. Despite ongoing advances, there is still a need for reliable and efficient clinical strategies [4,5]. In recent years, research has increasingly focused on preventing secondary damage, which has intensified interest in understanding the underlying mechanisms of secondary injury [5,6,7,8,9,10,11,12].

An SCI initiates a cascade of complex cellular responses, including inflammation, axonal degeneration, and cell death [13,14,15,16,17,18,19,20]. Among these, the spatiotemporal dynamics of cell death play a central role in the secondary injury pathophysiology and influence long-term neurological outcomes. While necrosis is characterized by uncontrolled cell membrane rupture and inflammation [16,21,22], apoptosis is a regulated, energy-dependent process that follows a regulated sequence [7,13,16,20,21,23]. Although apoptosis and necrosis may share some common pathways, their early post-injury patterns are often distinct and can independently affect the prognosis [5,7,9,15,16,18,21,24]. Understanding the spatial and temporal patterns of these death modalities is essential for identifying therapeutic windows and improving clinical outcomes.

Numerous studies have employed morphological or enzymatic markers, such as a TUNEL assay, caspase-3, and annexin-V, to detect apoptosis in SCI models. However, these markers often lack specificity or sensitivity in early-stage apoptosis or may overlap with necrotic signatures. Single-stranded DNA (ssDNA) immunostaining has emerged as a reliable alternative, offering specificity by detecting DNA denaturation, an early hallmark of apoptosis, without cross-reactivity with necrotic cells. Despite its utility, ssDNA immunostaining remains underutilized in experimental SCI research [25,26].

This experimental research reports an ssDNA antibody-based detection to evaluate the spatiotemporal distribution of apoptotic cells in a well-characterized rat model of moderate spinal cord contusion injury. By correlating the anatomical location and post-injury timepoint, we aim to provide a topographic map of apoptotic patterns within and adjacent to the injury site. To complement this, Cathepsin B, a lysosomal protease associated with necrotic pathways, was included in a descriptive capacity to aid the spatial interpretation of cell death dynamics and help contextualize regions that exhibited morphological signs of necrosis.

Our goal is to enhance understanding of localized cell death patterns post-SCI and support efforts to refine surgical or pharmacological interventions targeting the secondary injury phase.

## 2. Materials and Methods

### 2.1. Animals and Experimental Setup

This study was approved by the Animal Experimentations Ethics Board (05 D11 105 001) and carried out at Hacettepe University School of Medicine Surgical Research Laboratory (Ankara, Turkey). All procedures were conducted in accordance with the ARRIVE guidelines for reporting animal research [27]. A total of 36 adult male Wistar rats (200–300 g) were housed under controlled conditions (12-h light/dark cycle, 23 ± 1 °C) with free access to food and water. All animals were housed under identical conditions, cage positions were rotated weekly, and surgeries were performed in random order to minimize potential confounders. The animals were randomly assigned to trauma (n = 30) and control (n = 6) groups, the experimental unit being every individual Wistar rat. The trauma group was subdivided into six subgroups (n = 5/group), and the control and trauma groups were sacrificed at 1, 6, 24, 48, 72, and 168 h post-injury. These timepoints were selected to represent critical phases of secondary injury: 6 h for the acute phase of cell death initiation, 24 h to capture the peak of apoptosis, 48–72 h for progressive necrosis and early phagocytic responses, and 168 h (7 days) for the early subacute stage characterized by gliosis and remodeling while avoiding the transition into chronic repair phases [5,8,28]. The smaller number of animals in the control group reflects ethical considerations aimed at reducing animal use in compliance with the ARRIVE guidelines since the control values were consistent across the timepoints and served primarily as a baseline reference. Statistical power was ensured by using five animals per trauma subgroup, enabling reliable detection of spatial and temporal differences in cell death dynamics. From each rat, five 0.5 cm spinal cord segments were collected—one at the epicenter and two each from cranial and caudal segments—to yield a total length of 2.5 cm for the histological analysis. Control experimentals underwent laminectomies, and equivalent segments were harvested at matching timepoints.

### 2.2. Surgical Procedure

**Figure 1:**
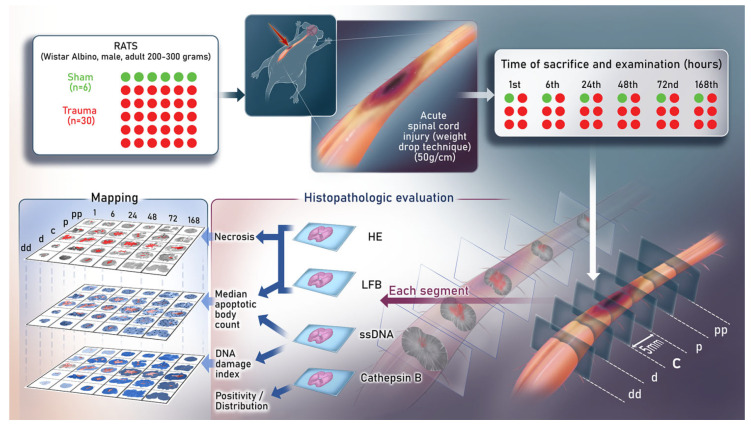
Experimental design, surgical procedure, and injury induction. A standardized moderate spinal cord contusion injury (50 g/cm) was produced at T9–T11 via a 5 g stainless steel rod dropped through a 10 cm vertical guide tube. The artist’s depiction shows intraoperative exposure of the spinal cord after laminectomy, the contusion setup, histopathologic processing, and digital mapping workflow. The mapping scheme illustrates the anatomical subdivision into five rostro-caudal segments (PP: proximal–proximal, P: proximal, C: epicenter/center, D: distal, DD: distal–distal) across six timepoints post-injury (1, 6, 24, 48, 72, 168 h), forming a 6 × 5 spatiotemporal grid. This figure was designed to serve as a graphical abstract, providing an integrated overview of study design and analysis.

Rats were anesthetized with intraperitoneal ketamine (Ketalar^®^ 5%, Eczacibasi Parke-Davis, Istanbul, Türkiye) (50 mg/kg) and xylazine (Rompun^®^ 2%, Bayer, Istanbul) (8 mg/kg) and were fixed to the experiment table in the prone position. After shaving and disinfecting the surgical area with povidone–iodine, a midline dorsal incision was made at the T9–T11 level. A laminectomy was carefully performed without disrupting the dura mater. Once the spinal cord and surrounding structures were confirmed to be intact, a standardized contusion injury (50 g/cm) was created using a 5 g stainless-steel rod dropped through a 10 cm vertical guide tube, targeting the center of the exposed cord according to a previously described drop model [6,29,30,31]. After achieving hemostasis, the muscle and skin layers were closed anatomically (Figure 1).

### 2.3. Postoperative Care

Postoperative care included recovery in a warmed cage with ad libitum food and water. Bladders were emptied every 12 h using the Crede maneuver. Gentamicin (Garamycin, Eczacibasi Schering-Plough, Istanbul, Türkiye) (0.2 mg/100 g/day) was administered for 7 days or until euthanasia.

### 2.4. Morphologic Analyses, Immunohistochemistry, and Rostro-Caudal Extension of the Lesion

At designated timepoints, the rats were transcardially perfused with pre-cooled saline and spinal cord tissue was fixed in 10% buffered formalin. A 2.5 cm spinal cord segment encompassing the injury epicenter and adjacent regions was divided into 0.5 cm blocks: epicenter (C), close rostral (P), distant rostral (PP), close caudal (D), and distant caudal (DD). Sections (6 µm) were stained with H&E (3 slides) and Luxol Fast Blue (2 slides) and immunostained using anti-ssDNA (IBL, #18731, 1:400, Fujioka, Japan,) and anti-Cathepsin B (Santa-Cruz SC-38333, clone CB131, 1:20, Dallas, TX, USA) antibodies. Additional immunostaining included GFAP (Novocastra, clone GA5, 1:400, Newcastle Upon Thyme, UK), CD68 (DAKO, Clone KP1, 1:10 000, Santa Clara, CA, USA), D2-40 (DAKO, clone D2-40, 1:40, Santa Clara, CA, USA), CD31 (DAKO, clone JC70A, 1:100, Santa Clara, CA, USA), and CD34 (DAKO, clone Qbend10, 1:40, Santa Clara, CA, USA) when required for a morphological differential. Staining was performed using an automated system (Ventana Benchmark XT immunostainer, Indianapolis, IN, USA), with detection using the iVIEW DAB Paraffin kit (760-091, Ventana, Indianapolis, IN, USA).

#### Quantification Approach, Digital Mapping, and Blinding

All immunostained sections were scanned at a high resolution using a NanoZoomer whole-slide scanner (Hamamatsu Photonics, Hamamatsu, Japan), which generated digitized photomicrographs for reliable detailed evaluation. These high-fidelity images derived directly from histological slides rather than schematic illustrations served as the foundation for identifying, quantifying, and mapping ssDNA-positive (apoptotic) cells and Cathepsin B-positive necrotic areas across anatomically defined spinal cord segments. Apoptotic cells, visualized as brown nuclei via ssDNA staining, were quantified by manual counting under 40× magnification and normalized against hematoxylin-stained (blue) non-apoptotic nuclei. Morphological verification was performed using serial H&E sections or complementary stains to ensure accuracy. This digital mapping approach enabled an accurate, reproducible, and anatomically resolved correlation of cell death patterns along the rostro-caudal axis, allowing for a more objective and transparent spatial analysis of post-injury apoptotic and necrotic events. To minimize the observer bias, all image quantification was performed by two independent investigators (A.A. and H.O.) blinded to the group allocation.

### 2.5. Statistical Analyses

Non-parametric tests were used considering the variable types, number of subjects, and data distribution. The Kruskal–Wallis test assessed the apoptosis across the timepoints. The Jonckheere–Terpstra test evaluated the trends across the lesion zones. Any *p*-values < 0.05 were considered significant. Analyses were performed using SPSS 16.0 (SPSS Inc., 2009 Chicago, IL, USA).

## 3. Results

### 3.1. Histopathological Examination Using H&E and LFB Histochemistry: Evaluation Using Injury Site, Necrosis Distribution, Edema, Chronologic Evaluation, and Mapping

**Figure 2:**
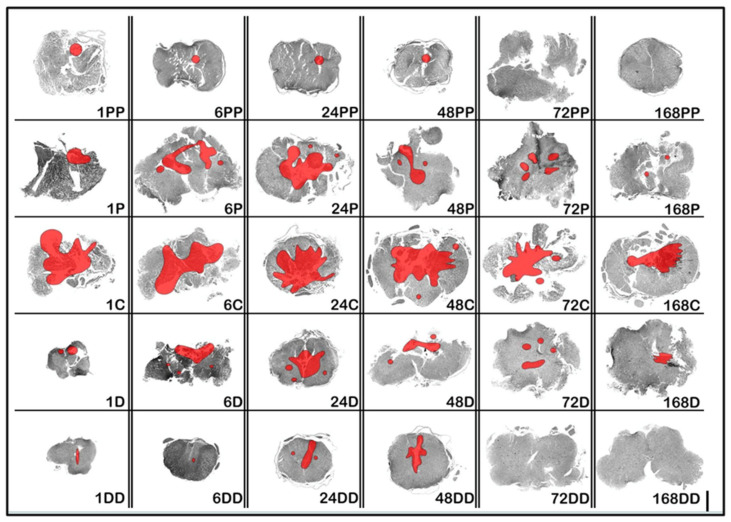
Spatiotemporal distribution of histopathological features and primary injury: necrosis mapping after the SCI. Representative H&E-stained spinal cord sections illustrate the progression of the hemorrhage, edema, and necrosis following injury. Early edema in the white matter and petechial hemorrhages in the gray matter appeared within 6–72 h post-trauma and transitioned into gliosis by day 7. Necrosis, confirmed morphologically and supported by Cathepsin B staining, peaked at the epicenter at 24–48 h. These necrotic areas are demarcated in red on real histological images and overlaid on subsequent topographic maps (also in Figure 3 and Figure 4) to contextualize the primary injury zones. This map is derived from real histological sections, showing the rostro-caudal extent and severity of the tissue disruption. Five anatomical segments are labeled as C (epicenter), P (proximal/close rostral), PP (distal/distant rostral), D (proximal/close caudal), and DD (distal/distant caudal), along with six timepoints (1–168 h). All images were obtained under identical magnification using a NanoZoomer whole-slide scanner.

Compared with the control sections, the injury epicenter exhibited a prominent hemorrhage, edema, and tissue disintegration starting from the first hour post-injury. These changes plateaued by 24–48 h and began to diminish by 72 h, with a marked reduction by 168 h. The hemorrhage and edema were consistently less severe in the rostral and caudal segments than at the epicenter throughout the time course. Early edema predominantly involved the white matter, while petechial hemorrhages were observed in the gray matter between 6 and 72 h. By day 7, reactive gliosis, including astrocytosis and microgliosis, became apparent. The region of primary damage, initially characterized by the hemorrhage, evolved into frank necrosis by 24–48 h, and associated edema extended into neighboring segments. Mapping of these histological alterations is visualized in Figure 2. Although Luxol Fast Blue staining revealed some vesicular axonal dissociation and granular axoplasmic degeneration, these findings were mild and not consistently distributed across the experimental groups.

### 3.2. Distribution of Apoptotic Cells Using ssDNA Immunohistochemistry: Evaluation of DNA Damage and Rostro-Caudal and Spatial Distributions

**Figure 3:**
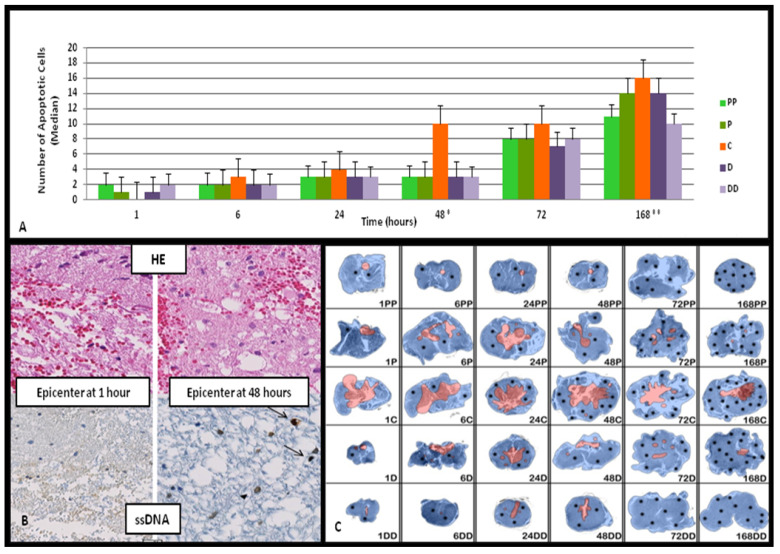
Apoptotic cell counts: spatial and temporal progression following an SCI. (**A**) Bar graph illustrating the median number of ssDNA-positive apoptotic cells at five anatomical levels (epicenter (c), proximal/distal rostral and caudal) across six timepoints. Statistically significant spatial differences were observed at 48* and 168** h (* *p* = 0.04, ** *p* = 0.004, Kruskal–Wallis test). (**B**) Representative histological images showing H&E and ssDNA staining at the epicenter (1 h and 48 h), with apoptotic cells marked by arrows (magnification ×400). (**C**) A composite digital map visualizing the apoptotic cell distribution. Necrotic zones (from Figure 2) are marked in red. Black dots scaled to median apoptotic counts per segment demonstrate the topographic spread of apoptotic cell counts of ssDNA, illustrating temporospatial changes from acute (1–24 h) to subacute (48–168 h) stages. The mapping was performed on real digitized microphotographs obtained under identical magnification using a NanoZoomer whole-slide scanner and are not schematic illustrations. Regional differences between the epicenter and perilesional zones are highlighted. Data were analyzed using Kruskal–Wallis and Jonckheere–Terpstra trend tests; *p*-values are reported in the text and the figure panels.

The distribution of ssDNA-positive apoptotic cells was evaluated across defined spinal cord segments (epicenter, close and distant rostral, and close and distant caudal) and timepoints (1 to 168 h post-injury). Data are presented in two complementary ways, namely, spatial mapping at each timepoint (Figure 3A) and temporal progression at each anatomical level (Figure 3B), to avoid conflation between location-based and time-based analyses.

At 1-h post-injury, no apoptotic cells were detected at the epicenter. However, apoptotic nuclei were observed in the rostral and caudal segments, particularly at more distant levels. Apoptosis increased in all regions from 6 to 48 h and peaked at 168 h. The epicenter showed a delayed onset of apoptosis, with a notable increase beginning at 24 h and a sharp rise between 48 and 168 h. In contrast, peripheral rostral and caudal zones displayed a more gradual increase that started earlier, between 6 and 48 h.

Statistical analysis (Kruskal–Wallis test) revealed significant spatial variation at both 48 h (*p* = 0.004) and 168 h (*p* = 0.0004), indicating that at least one segment differed significantly from the others in the apoptotic cell count at these timepoints (Figure 3A).

When the data were reorganized to assess the temporal evolution at each specific level, the apoptotic counts increased steadily from 1 to 168 h. The Jonckheere–Terpstra test confirmed a significant upward trend across timepoints for each segment (*p* < 0.001). While all regions exhibited progressive increases, the epicenter showed delayed but ultimately higher apoptotic activity compared with the adjacent zones. Apoptotic nuclei were more prominent in the white matter and were occasionally seen in the vascular endothelia and neurons, though predominant in the glial cells. Spatial mapping using real digitized microphotographs (Figure 3B) that were quantitatively combined with the changes that reflected the primary DNA damage findings from the above paragraph confirmed lateral and dorsal predominance of apoptosis, which became more extensive over time. The density of apoptotic bodies at each level is depicted as scaled black dots in Figure 3C.

### 3.3. DNA Damage Index and Temporal and Rostro-Caudal Distributions

While the raw apoptotic cell counts provided valuable spatial insights, they may be influenced by concurrent pathological processes, such as edema, hemorrhage, or tissue loss, that alter the cellular density. To correct for these confounding factors, we calculated a DNA damage index defined as the proportion of ssDNA-positive nuclei to the total number of nuclei in each cross-sectional area. This apoptotic index offers a normalized metric for assessing the apoptotic burden relative to total cellularity, allowing a more accurate interpretation of the injury severity and apoptotic dynamics.

**Figure 4:**
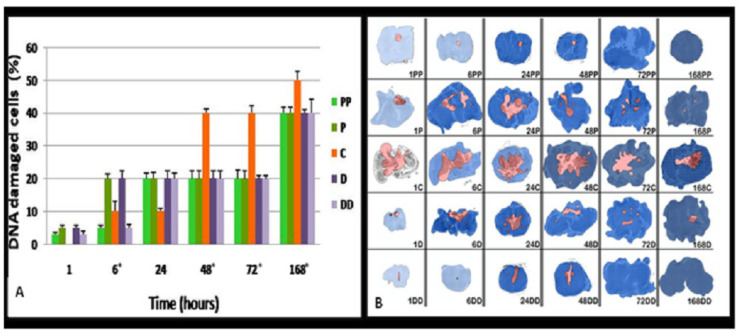
Apoptotic index (DNA damage percentage): normalized mapping of lesion severity. (**A**) Spatial profile of the apoptotic index—ratio of the ssDNA-positive nuclei to the total nuclei—across injury levels over time. Significant differences were seen at 6*, 48*, 72*, and 168* h (Kruskal–Wallis * *p* < 0.05). (**B**) Composite color-coded topographic map performed on real digitized microphotographs obtained under identical magnification using a NanoZoomer whole-slide scanner integrates necrotic zones (red overlay from Figure 2) with the DNA damage index. Blue color intensity and halftone meshing correspond to percentage of apoptotic nuclei in each region. All data were derived from real high-resolution histological sections. The data were analyzed using Kruskal–Wallis and Jonckheere–Terpstra trend tests; *p*-values are reported in the text and the figure panels.

Figure 4A displays the spatial profile of the apoptotic index across the epicenter, rostral, and caudal spinal segments at different timepoints. At the epicenter, the index remained relatively stable from 6 to 24 h, then rose sharply at 48 h, and plateaued between 48 and 72 h, ultimately peaking at 168 h. In the rostral and caudal regions, the apoptotic index showed an earlier rise, with elevated values already present by 6 h and sustained through to 72 h, eventually reaching near-epicentral levels by 168 h. Statistical comparisons showed significant spatial differences in apoptotic index values at 6, 48, 72, and 168 h (*p* = 0.02, 0.0015, 0.007, and 0.0176, respectively), while no difference was found at 24 h (*p* = 0.64), suggesting transient synchronization of the damage burden across the levels during this interval. Regarding the temporal progression of apoptotic index values within each anatomical spine region, all segments displayed a biphasic increase: an initial phase from 6 to 24 h, followed by a steeper rise from 48 to 72 h. Peak values were consistently recorded at 168 h. Notably, the peripheral segments (PP and DD) showed early increases in the apoptotic index that paralleled or even preceded changes at the epicenter. These patterns reflect both localized and propagating apoptotic activity within the injured spinal cord.

The combined spatial and temporal mapping of the apoptotic index is visualized in Figure 4B. Blue intensity and halftone shading correspond to the percentage of DNA-damaged cells at each segment, highlighting the progressive rostro-caudal spread of the apoptotic burden and validating the normalized metric values for lesion characterization.

### 3.4. Cathepsin B Immunohistochemistry

**Figure 5:**
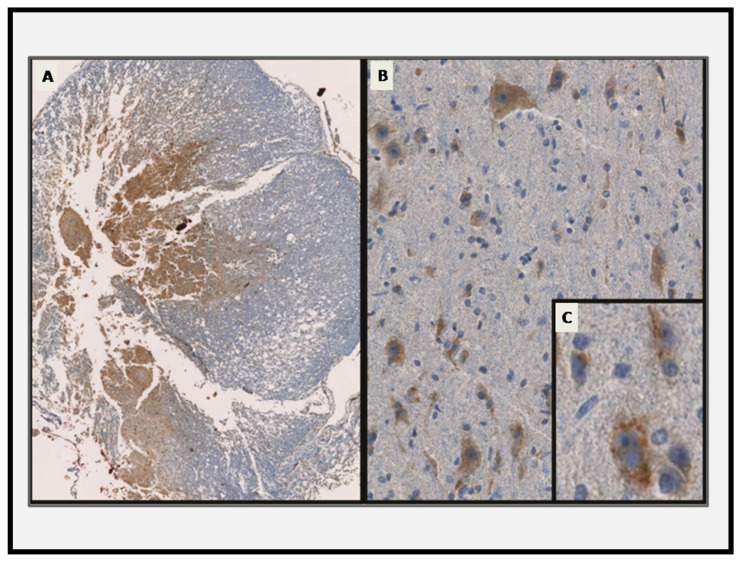
Immunohistochemical analysis of Cathepsin B expression post-SCI. (**A**) At the epicenter, diffuse and irregular cytoplasmic Cathepsin B staining was observed within gray and adjacent white matter by 48 h (magnification ×50). (**B**) At 168 h, the Cathepsin B staining returned to a fine granular pattern within neurons and glia, consistent with a restored lysosomal distribution (magnification ×40). (**C**) Inset at higher magnification (×1000) shows cytoplasmic localization. These observations support early necrotic activity and late-stage cellular recovery.

In the control group, immunohistochemical analysis of Cathepsin B showed fine granular staining, suggesting a regular lysosomal distribution of Cathepsin B. However, in the trauma groups, after the injury induction, at the epicenter, mostly gray and partly white matter showed a diffuse irregular staining that disappeared at 72 h; at 168 h, neurons and glia contained Cathepsin B cytoplasmic staining (Figure 5).

## 4. Discussion

The present study aimed to elucidate the chronological and topographical evolution of cell death (apoptosis and necrosis) following SCI using histopathological and immunohistochemical techniques in a moderate contusion rat model. By combining ssDNA and Cathepsin B expression analyses, we provide insights into the molecular and structural pathology of SCI.

An SCI is a devastating condition with lasting physical, psychological, and socioeconomic impacts. Despite extensive research, there remains no universally effective treatment. The injury initiates with a primary mechanical insult and progresses into a secondary cascade involving ischemia, oxidative stress, excitotoxicity, inflammatory mediators, and programmed cell death [5,7,8,9,32,33]. This secondary injury is believed to contribute substantially to long-term neurological deficits and has thus become a key therapeutic target [4,34].

Experimental models, particularly contusion-based injuries, closely simulate a human SCI and have been widely adopted for preclinical studies [35,36]. Apoptosis is a prominent feature of secondary injury and has been traditionally detected using TUNEL, caspase-3, or annexin-V assays. However, these methods suffer from specificity limitations or require fresh tissue [37,38,39]. By contrast, ssDNA immunostaining selectively labels DNA denaturation, an early marker of apoptosis, without cross-reactivity to necrosis, offering both specificity and stability in formalin-fixed tissue [25,26,40]. Despite its proven utility in oncology and forensic medicine, ssDNA remains underutilized in SCI research [41,42,43].

The H&E and LFB-based morphological maps (Figure 2) confirmed a hemorrhage and necrosis at the epicenter as early as 1-h post-injury, with edema spreading longitudinally from the lesion core. This histopathological progression underpins the secondary damage hypothesis. ssDNA staining further revealed that apoptotic DNA damage was absent at the epicenter during the first hour but was detectable at peripheral sites, suggesting that a severe mechanical insult may induce necrosis, while a milder peripheral injury initiates apoptosis.

By 6–24 h, the numbers of apoptotic cells increased along the rostral and caudal segments and began to rise at the epicenter by 24 h. A marked peak was noted at 48 h in the center, consistent with the onset of subacute injury. This wave of apoptosis extended outward, reaching distal regions by 72–168 h. These patterns are statistically validated by both median apoptotic cell counts and index data (Figure 3 and Figure 4).

Importantly, the apoptotic index, which normalizes apoptotic cell counts to the total cell number, allowed for a more accurate interpretation of tissue burden that accounted for density changes due to edema or necrosis. This metric demonstrated an early rise in peripheral areas and a delayed central peak, reinforcing the notion that apoptosis spreads outward from the injury core.

A notable morphological finding was that apoptosis predominantly affected the glial and endothelial cells, with relatively lower involvement of neurons. This aligns with the concept that necrosis predominates in neurons exposed to direct trauma, while glial cells, particularly oligodendrocytes, undergo programmed cell death [4]. Our immunohistochemistry-based approach offers spatial localization superior to prior biochemical assays.

Cathepsin B expression patterns were also informative. In the control tissue, Cathepsin B was localized to neuronal cytoplasm. Post-injury, its diffuse expression at the epicenter diminished by 72 h and reappeared intracellularly in microglia at 168 h, possibly reflecting phagocytic uptake. This aligns with earlier findings implicating Cathepsin B in lysosomal rupture and necrotic pathways [34,44,45]. Although our evaluation was descriptive, the temporal disappearance and reappearance of Cathepsin B may mark distinct necrotic and reparative phases.

Our findings highlight ssDNA as a robust, reliable, and specific marker for mapping apoptosis, supporting its broader adoption in SCI models [25,26,46]. Clinically, the centripetal spread of apoptosis from peripheral to central regions suggests that therapies directed solely at secondary injury may be insufficient; the primary lesion core and secondary cascades should be targeted. Since central apoptosis peaks after 48 h and glial responses extend into the subacute phase, treatment evaluation should extend beyond the 48 h window. Effective management may therefore require a combined strategy: early pharmacological interventions addressing secondary injury, together with simultaneous approaches targeting the primary lesion. These results align with recent studies implicating XIAP, hydrogen-rich saline, and mitochondrial DNA–cGAS–STING signaling, underscoring the need for temporally and spatially informed therapeutic strategies for SCIs [47,48,49,50].

This study has several limitations. We did not perform parallel validation using additional apoptotic markers, such as cleaved caspase-3, Annexin V, or TUNEL, alongside the ssDNA immunostaining. This methodologic limitation raises the possibility of false positive results in regions of extensive necrosis. However, the application of strict morphological criteria and the nuclear, rather than cytoplasmic, localization of ssDNA reduce the likelihood of such a misinterpretation.

Although we detected ssDNA positivity primarily in glial cells by utilizing cell-specific markers, such as GFAP, CD68, D2-40, CD31, and/or CD34, on adjacent sections to assist with the morphological differentiation, in the subset of cases when the cell identity was unclear, this approach is inherently limited compared with double-labeling techniques. Future studies incorporating co-localization strategies would be more effective at distinguishing cell types and further elucidating cellular dynamics of secondary injury. Additionally, our interpretation that gliosis was the predominant outcome of necrosis resolution is based largely on the morphological assessment. While CD68 was used to identify phagocytic cells, the absence of dual labeling and the difficulty in visualizing overlapping reactivity limited our ability for definite confirmation. These factors should be considered when interpreting tissue clearance dynamics in the injured spinal cord.

The present study focused on histopathological and immunohistochemical parameters without including functional or behavioral outcomes. Incorporating motor function evaluations in future studies would significantly strengthen the translational value and provide a more comprehensive understanding of the relationship between histological changes and clinical recovery.

Finally, while ssDNA immunostaining was quantified through a nuclear counting approach and digital mapping, Cathepsin B analysis remained primarily qualitative. Future studies incorporating fully quantitative assessment of Cathepsin B expression, alongside chronic stage evaluation and inflammatory biomarker profiling, would provide stronger mechanistic support.

Despite these limitations, our study demonstrates that apoptosis evolves in a temporo-spatially organized manner after an SCI, with peripheral initiation and delayed central amplification. ssDNA-based mapping reveals consistent, quantifiable apoptotic trends, while Cathepsin B highlights necrotic zones. These results enhance our understanding of secondary injury mechanisms and may inform therapeutic targeting of specific injury phases in an SCI.

## 5. Conclusions

This study provides a detailed temporospatial characterization of apoptotic and necrotic cell death in a moderate spinal cord contusion model using ssDNA and Cathepsin B immunohistochemistry alongside histopathological mapping. By distinguishing DNA damage from overt cell death and quantifying apoptotic indices, we established a reproducible framework for identifying progression phases of secondary injury. Our findings highlight that apoptosis originates in peripheral segments and propagates toward the epicenter, while necrotic changes dominate the lesion core early.

The selective use of ssDNA allowed for sensitive detection of early apoptotic cells, particularly in glial and endothelial populations, with accurate spatial correlation free of necrotic confounding. Cathepsin B expression, though assessed qualitatively, further supported regional necrotic dynamics and potential phagocytic responses.

Collectively, our results reinforce the importance of nuanced markers in spinal cord injury research and suggest that temporally targeted interventions should account for the timing and spatial distribution of apoptosis and necrosis. Future studies incorporating broader biomarker panels and functional outcomes will be critical to translate these insights into therapeutic applications.

## Data Availability

The data presented in this study are available upon request from the corresponding author.
